# Prevalence of ineffective breastfeeding techniques and its associated factors among breastfeeding mothers in Ethiopia: A systematic review and meta-analysis

**DOI:** 10.1371/journal.pone.0303749

**Published:** 2024-06-13

**Authors:** Gizachew Yilak, Befikad Derese Tilahun, Biruk Beletew Abate, Alemu Birara Zemariam, Eyob Shitie Lake, Mulat Ayele, Alemayehu Sayih Belay

**Affiliations:** 1 Department of Nursing, College of Health Sciences, Woldia University, Woldia, Ethiopia; 2 Department of Midwifery, College of Health Sciences, Woldia University, Woldia, Ethiopia; 3 Department of Nursing, College of Health Sciences, Wolktie University, Wolktie, Ethiopia; University of Gondar, ETHIOPIA

## Abstract

**Background:**

Poor attachment, suckling, and positioning contribute to ineffective breastfeeding techniques. Poor weight gain, stunting, and decreased immunity are caused by insufficient breast milk intake owing to ineffective breastfeeding techniques. Numerous primary studies conducted in Ethiopia have revealed negative effects of ineffective breastfeeding techniques. However, inconsistencies have been observed among these studies, and no review has been conducted to report the amalgamated magnitude and associated factors. Therefore, this review aimed to estimate the national prevalence and factors associated with ineffective breastfeeding techniques in Ethiopia.

**Methods:**

Using PRISMA standards, we systematically reviewed and meta-analyzed articles from PubMed, Cochrane Library, and Google Scholar to investigate the prevalence and associated factors of ineffective breastfeeding techniques. Q and I^2^ tests were used to assess heterogeneity across studies. To evaluate the national prevalence and effect size of the linked covariates, a weighted inverse variance random-effects model was used. Subgroup analysis was performed based on the region, sample size, and year of publication. Funnel plots and Egger’s regression tests were used to examine publication bias. A sensitivity analysis was also performed to determine the impact of the studies.

**Results:**

The analysis included a total of eleven studies with 4,470 participants used in the analysis. The pooled prevalence of ineffective breastfeeding techniques in Ethiopia was 53.13% (45.49, 60.78) no formal education (AOR = 3.42; 95% CI:1.67–5.18; I^2^ = 72.2%; P = 0.0132), primipara (AOR = 2.72;95% CI:1.81–3.64; I^2^ = 46.7%; P = 0.131), postnatal care (PNC) (AOR = 1.84; 95% CI:1.35–2.32; I^2^ = 0%; P = 0.53), breastfeeding counseling (AOR = 1.93; 95% CI:1.23–2.63; I^2^ = 0%;P = 0.35), home delivery and having breast problem (AOR = 3.11; 95% CI:1.09–5.16; I^2^ = 0%;P = 0.877) and (AOR = 2.22; 95% CI:1.28–3.16; I^2^ = 0%;P = 0.80) respectively were significantly associated with ineffective breastfeeding techniques.

**Conclusion:**

The prevalence of ineffective breastfeeding techniques in Ethiopia remains high. Policymakers and program officials should focus on empowering women, increasing institutional delivery, and providing counseling on breastfeeding throughout the maternal continuum of care. These measures will improve breastfeeding techniques and lead to better health of both infants and women.

## Introduction

The best nutrition for infants is breast milk, which has an optimal ratio of vitamins, proteins, fats, and antibodies [1–3]. Proper breastfeeding is essential for mothers to help with involution (4) and lower their risk of developing breast cancer [4], a risk factor for osteoporosis, ovarian epithelial cell cancer, and coronary artery disease (CAD) [5–7]. Achieving universal proper breastfeeding can avert 20,000 breast cancer-related deaths and 823,000 deaths in children under five per year [8]. However, most mothers were unaware that breastfeeding is a taught skill that requires practice and patience [9].

The three components of breastfeeding techniques are the position, attachment, and sucking. The method of holding the baby in relation to the mother’s body is referred to as positioning. Sucking is the act of drawing milk from the nipple into the mouth, and attachment determines whether the infant has enough areola and breast tissue in the mouth [10]. Poor positioning, attachment, and suckling are components of ineffective breastfeeding techniques (IBT), which result in insufficient intake of breast milk, leading to poor weight gain, stunting, and immune decline [9, 11].

Inappropriate positioning of the baby and attachment cause lactating women to experience a number of issues. Evidence shows that 58% of lactating mothers with poor breastfeeding techniques are exposed to early weaning from exclusive breastfeeding because of persistent nipple pain. This pain affects mental stability, making mothers psychologically distressed and interfering with their normal activities and ability to bond with their children. The interruption of breastfeeding results in serious acute infectious diseases, such as diarrhea and acute respiratory infections, and chronic diseases, such as diabetes, diminished mental ability, and an increased risk of epileptic disorders during infancy [12, 13].

According to the 2019 Mini-Ethiopian Demographic Health Survey (MEDHS), 37%, 21%, and 7% of children under the age of five are stunted, underweight, and wasted, respectively [14]. Breast engorgement, impaired nipples, mastitis, and breast abscesses are caused by poor breastfeeding techniques [13, 15]. Despite a dramatic decline in child mortality worldwide over the past three decades, approximately 5.3 million children under the age of five died in 2018; about 50 percent of these deaths are attributable to sub-Saharan Africa [16]. Suboptimal breastfeeding techniques increase the risk of infant mortality; non-breastfed and partially breastfed newborns are 14 and 3 times more likely to die in the first six months than exclusively breastfed infants. Furthermore, inappropriate positioning contributes to diarrhea and respiratory illnesses [12, 17]. Poor breastfeeding techniques leads to a decline in the use of exclusive breastfeeding (EBF) [15].

Despite progress over the last 14 years, only 59.0% of infants under the age of six months in Ethiopia have received EBF [14]. The prevalence of ineffective breastfeeding techniques ranges from 30 to 70 percent in Denmark, Brazil, Nepal, India, Libya, and Ethiopia [13, 18–21]. Breast problems such as lack of maternal education, breastfeeding experience, home delivery, inadequate counseling, operational deliveries, and primiparity are all variables that contribute to ineffective breastfeeding techniques [11, 13, 18, 22]. Although there is evidence that successful breastfeeding techniques benefit both the mother and her child in the short and long term, breastfeeding techniques are not well established in underdeveloped nations [23]. IBT and their associated determinants have been studied primarily in underdeveloped countries, including Ethiopia [13, 18–21]. However, the reported findings are inconsistent and, to the best of our knowledge, no systematic review or meta-analysis has been conducted to address these conflicting results in Ethiopia. Therefore, this systematic review and meta-analysis aimed to examine the prevalence of ineffective breastfeeding techniques and identify the associated factors among breastfeeding mothers in Ethiopia.

## Methods

### Reporting

The findings of this review are presented in accordance with the Preferred Reporting Items for Systematic Review and Meta-Analysis statement recommendations (S1 Table).

### Searching strategy and information sources

We found papers from PubMed, the Cochrane Library, and Google Scholar that provided data on the prevalence of and potential risk factors for IBFT in Ethiopia. To retrieve additional publications, the search used Medical Subject Headings (MeSH) terms and keywords, combinations, and snowball searching in the reference list of papers found through the database search. Articles with missing or incorrect data were resolved by contacting the corresponding authors. Unpublished studies were obtained from the official websites of international and local organizations’ and universities.’

### Medical Subject Headings (MeSH) terms and keywords were used to conduct the search

"(Prevalence OR (Prevalence[MeSH Terms]) magnitude OR magnitude [MeSH Terms]) or epidemiology) OR (epidemiology [MeSH Terms]) AND (causes OR (causes [MeSH Terms]) determinants OR (determinants [MeSH Terms]) (related factors) OR (related factors [MeSH Terms]) OR predictors OR (predictors[MeSH Terms]) OR (risk factors) OR (risk factors [MeSH Terms]) OR (poor breastfeeding techniques OR (poor breastfeeding techniques [MeSH Terms]) (ineffective breastfeeding techniques OR (ineffective breastfeeding techniques [MeSH Terms]) OR (position, attachment, and suckling OR (position, attachment, and suckling [MeSH Terms]) OR (effective breastfeeding techniques) OR (effective breastfeeding techniques [MeSH Terms]) AND (Ethiopia)" (S1 File) Moreover, the searching strategy that had been used for Google Scholar was illustrated (S2 File). The searching date was until November 12, 2023.

We also searched the reference lists of the remaining papers to identify new studies relevant to this review. The criteria for study selection and eligibility to remove duplicate studies were exported to the reference manager program EndNote version 8. Before retrieving full-text publications, two investigators (GY and ASB) independently assessed the selected studies using their titles and abstracts. We further screened the full-text papers using the prespecified inclusion criteria. Disagreements regarding the final selection of studies to be included in the systematic review and meta-analysis were discussed with additional reviewers (MA, BD, BBA, and ESL) during a consensus meeting.

### Inclusion and exclusion criteria

All observational (cross-sectional) studies were included in this meta-analysis. Research published in English between 2010 and 2023 in Ethiopia has examined the prevalence of ineffective breastfeeding techniques among breastfeeding mothers and/or one or more associated factors. Unpublished studies on breastfeeding mothers have also received attention. The analysis did not include editorials, anonymous reports, qualitative studies, or citations without an abstract or full text. Moreover, studies that failed to disclose noteworthy findings were excluded. Breastfeeding mothers receiving postnatal care or visiting the child vaccination department during the data collection period met the inclusion and exclusion criteria of the included studies. A severely ill baby whose mother or other caregiver declined to include in the study was not allowed.

### Quality assessment

After integrating the database search results, duplicate articles were deleted by using EndNote (version X8). The quality appraisal checklist developed by the Joanna Briggs Institute (JBI) was employed [24, 25]. The quality of the studies was evaluated by four independent writers. Appraisal was repeated using trading notes. As a result, one study was evaluated by two authors. Any disagreement between the reviewers was resolved by averaging the scores. Studies were considered low risk or good quality if they scored 5 or higher for all studies and were included [24, 25]. However, studies with a score of 4 or lower were considered high risk or poor quality and were not included.

### Data extraction

The data extraction form that the authors created was an Excel file containing the following information: name of the author, year of publication, research region, study design, sample size, prevalence of IBT, and categories of factors that were reported. Four papers were randomly selected to test the data-extraction sheet. The extraction form was modified after the experiment using the template. Two authors used an extraction form to extract the data. The accuracy of the data was verified separately by third, fourth, and fifth authors. If necessary, discussions with a third and fourth reviewer helped settle any disputes that arose among the reviewers. Cross-referencing the data with the included papers allowed the correction of data errors. If incomplete data were received, the study was excluded after two email attempts to contact the corresponding author.

### Outcome measurement

An ineffective breastfeeding technique (IBT) was considered when a composite variable of the three constructs (position, attachment, and suckling) was present; therefore, lactating women with at least one of the constructs categorized as poor would be ineffective breastfeeding techniques [26, 27].

### Statistical analysis

After extracting the data in Microsoft Excel format, we loaded it into STATA version 17.0, a statistical software for further analysis. Standard error was computed for each study using a binomial distribution formula. A random-effects meta-analysis was used to pool the overall magnitude of ineffective breastfeeding techniques [28]. Forest plots were used to display the pooled prevalence of poor breastfeeding techniques with 95% confidence intervals (CI) and odds ratios (OR) with 95% CI to illustrate the factors related to ineffective breastfeeding techniques. Using p-values, inverse variance (I^2^) and Cochran’s Q statistics (Chi-square) were used to examine the heterogeneity among the studies [29].

In this study, an I^2^ value of zero indicated true homogeneity, whereas values of 25, 50, and 75% represented low, moderate, and high heterogeneity, respectively [30, 31]. We conducted a random effects model analysis of the data identified as heterogeneous. In addition, subgroup analysis was performed according to study region, sample size, and year of publication. When statistical pooling is not possible, non-pooled data are presented in a table form. Sensitivity analysis was employed to determine the effect of a single study on the overall estimation. Publication bias was checked using funnel plots and more objectively using Egger’s regression test [32].

## Results

**Fig 1:**
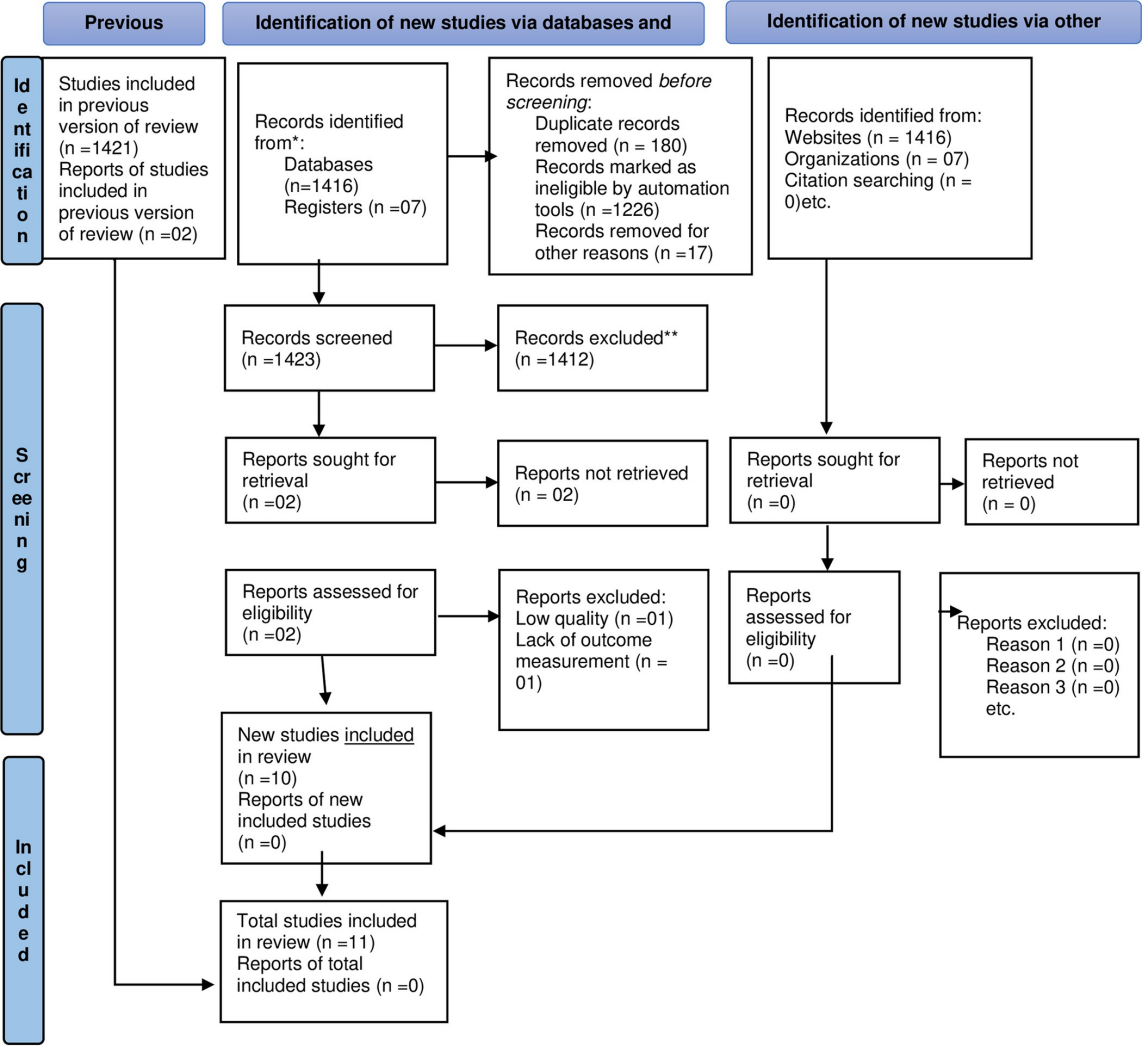
PRISMA flow chart for the selection of a systematic review and meta-analysis of ineffective breastfeeding techniques and their associated factors in Ethiopia.

Study selection: A total of 1423 studies were identified using electronic searches (through databases searching after removing duplicates, a total of 1423 studies were retrieved that were conducted from 2010 to 2023, of which 1394 were rejected by reading only the titles. Of the remaining 29 studies, 1^2^ were excluded after reading the abstracts. Finally, 17 studies were screened for full-text review, and 11 articles with (n = 4,470 study participants) were selected for the prevalence and/or associated factors of ineffective breast-feeding technique analysis (Fig 1).

### Characteristics of included studies

Table 1 summarizes the characteristics of the 11 included studies in the systematic review and meta-analysis [20, 26, 33–41]. Six studies were found in the Amhara region [26, 33, 35, 36, 38, 40], three in southern nation nationalities [34, 37, 39], one Harar city [20], and one in Addis Ababa city [41]. Most studies were published between 2016 and 2021. The studies included 252 [34] to 760 [33] participants (Table 1).

**Table 1:** Distribution of studies on the prevalence and determinants of ineffective breastfeeding techniques among breastfeeding mothers, 2010–2023.

Author/Reference	year	Region	study design	sample size	p	Quality score
Degefa, N. et al. [34]	2018	SNNPS	cross-sectional	252	61.51	7/8
Muche, A.A et al. [25]	2022	Amhara	cross-sectional	760	57.10	6/8
Safayi, B.L.et al. [26]	2021	Amhara	cross-sectional	410	48.00	7/8
Tiruye, G *et al. [20]	2018	Harar city	cross-sectional	412	43.40	6/8
Asmamaw, D.B. et al. [33]	2023	Amhara	cross-sectional	389	66.80	7/8
Desse, H. et al. [35]	2023	Amhara	cross-sectional	420	54.30	7/8
Yilak, G. et al. [39]	2020	SNNPS	cross-sectional	414	63.30	7/8
GELILA MEKURIY, Z.A. et al. [41]	2020	Addis Ababa	cross-sectional	253	59.70	5/8
HIRUT, Y. et al. [36]	2022	Amhara	cross-sectional	359	53.80	6/8
Wetete et al. [38]	2021	Amhara	cross-sectional	417	57.00	6/8
Tamiru, D et al. [37]	2016	SNNPS	cross-sectional	384	20.10	7/8

**Fig 2:**
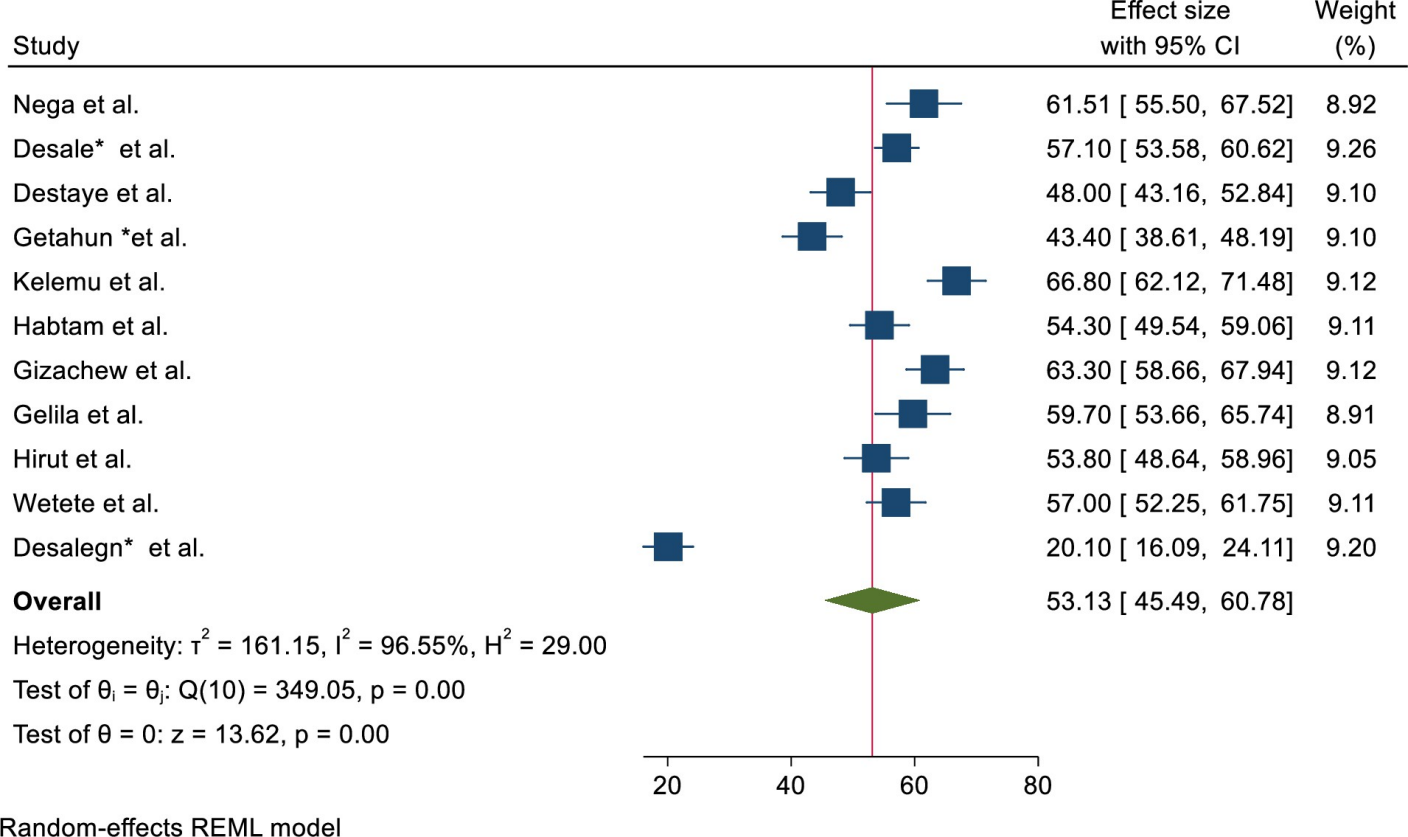
Frost plot on the pooled prevalence of ineffective breastfeeding technique among breastfeeding mothers in Ethiopia.

Meta-analysis Prevalence of ineffective breastfeeding techniques among breastfeeding mothers in Ethiopia: Most of the studies (n = 11) reported the prevalence of ineffective breastfeeding techniques [20, 33–40]. The prevalence of ineffective breastfeeding techniques was ranged from 20.1% [33] up to 67% [40]. The random-effects model analysis from those studies revealed that the pooled prevalence of ineffective breastfeeding techniques among breastfeeding mothers in Ethiopia was 53.13% (95% CI; 45.49–60.78; I^2^ = 97.1%; p < 0.001) (Fig 2).

### Subgroup analysis of the prevalence of ineffective breastfeeding techniques in Ethiopia

Subgroup analysis was performed by stratification according to region, sample size, and publication year. Based on this, the prevalence of ineffective breastfeeding techniques among breastfeeding techniques was found to be 53.13% (95%CI; (45.49–60.78) (Fig 2). Based on the study region, the prevalence of ineffective breastfeeding techniques among breastfeeding mothers was found to be 43.21% (40.51–45.92) in South nation and nationality, 56.43% (54.59–58.27) in Amhara region, 43.4% (38.61–48.19) in Harar City, and 59.7% (53.66–65.74) in Addis Ababa City (S1 Fig and Table 2).

**Table 2:** Subgroup analysis of the prevalence of ineffective breastfeeding techniques among breastfeeding mothers in Ethiopia by region, sample size, and year of publication.

Variables	Characteristics	Pooled prevalence (95% CI)	I^2^ (P value)
By region	Southern Nations, Nationalities, and Peoples’	43.21(40.51–45.92)	99.1
	Amhara	56.43(54.59–58.27)	84.6
	Harar	43.40(38.61–48.19)	-
	Addis Ababa	59.70(53.66–65.74)	-
By year of publication	2010–2018	36.30(33.57–39.04)	98.6
	2019–2021	56.88(54.39–59.38)	85.8
	2022–2023	58.04(55.85–60.23)	84
By sample size	Sample size <400	48.17(45.94–50.40)	98.6
	Sample size > = 400	54.36(52.54–56.18)	88.8

Based on the study sample sizes, the prevalence of ineffective breastfeeding techniques among breastfeeding mothers was found to be 48.17% (45.94–50.40) in sample size less than 400, while it was 54.36% (52.54–56.18) in sample sizes greater than 400 (S2 Fig and Table 2). Moreover, Based on the year of publication, the prevalence of ineffective breastfeeding techniques among breastfeeding was found to be 36.3% (33.57–39.04) from 2010 to 2018, 56.88% (54.39–59.38) from studies conducted from 2019 to 2021, and 58.04% (55.85–60.23) from studies conducted from 2022 to 2023 (S3 Fig and Table 2).

### Sensitivity analysis

We employed a leave-one-out sensitivity analysis to identify the potential source of heterogeneity in the analysis of the prevalence of ineffective breastfeeding techniques. The results of sensitivity analysis showed that our findings were not dependent on a single study. Our pooled estimated prevalence of ineffective breast-feeding techniques varied between 51.77% (43.07–60.46) [40] and 56.45%(52.12–60.79) [37] after deletion of a single study (Table 3).

**Table 3:** Sensitivity analysis of the prevalence of ineffective breastfeeding techniques among breastfeeding mothers in Ethiopia.

Study ommited	Coef.	[95% conf. Interval]	
Nega et.al	52.319782	43.368595	61.270973
Desale et.al	52.746006	43.262867	62.229141
Destaye et.al	53.659634	44.446472	62.872795
Getahun et.al	54.119324	45.037674	63.200977
Kelemu et.al	51.770378	43.078579	60.462173
Habtam et.al	53.029488	43.781063	62.277908
Gizachew et.al	52.123589	43.182449	61.064728
Gelila et.al	52.498497	43.503036	61.493954
Hirut et.al	53.079357	43.892124	62.266594
Wetete et.al	52.758598	43.558258	61.958935
Desalegn et.al	56.457226	52.116669	60.797779
Combined	53.131173	45.496486	60.782861

### Publication bias

Egger’s regression test p-value was 0.355, which indicated the absence of publication bias (S4 Fig). Out of the total included studies 06 studies [20, 26, 35, 39–41] revealed the factors associated with ineffective breastfeeding techniques among breastfeeding mothers (Table 4).

**Table 4:** Factors associated with ineffective breastfeeding techniques in Ethiopia.

Variables	Odds ratio(95%CI)	Author (reference)	Year	Pooled AOR(95%CI)	I^2^ (P-value)
No formal education	2.3(0.9–3.70)	Getahun [21]	2018	2.802(1.996–3.608	72.2%
	5.88(3.75–8.045)	Kelemu [33]	2023		
	2.1(0.95–3.25)	Habtam [27]	2023		
	5.00(0.9–9.10)	Gizachew [32]	2020		
Being primipara	3.3(2.45–4.15)	Getahun [21]	2018	2.76(2.16–3.358)	46.7%
	4.34(1.28–7.39)	Kelemu [33]	2023		
	2.5(1.05–3.95)	Habtam [27]	2023		
	1.8(0.7–2.9)	Gizachew [32]	2020		
Post-natal care	5.9(1.00–12.80)	Getahun [21]	2018	1.835(1.346–2.325)	53%
	2.1(0.85–3.350)	Habtam [27]	2023		
	2.50(0.60–4.40)	Gizachew [32]	2020		
	1.7(1.145–2.255)	Gelila [41]	2020		
BF couselling	4.94(1.83–13.36)	Kelemu [33]	2023	1.934(1.237–2.632)	43.5%
	1.7(0.85–2.55)	Getahun [21]	2018		
	2.3(1.4–3.9)	Gizachew [32]	2020		
home delivery	2.85(1.22–6.66)	Desale [25]	2022	3.108(1.095–5.12)	87.7%
	3.02(1.12–8.14)	Kelemu [33]	2023		
	4.5(1.6–13.1)	Gizachew [32]	2020		
breast problem	4(1.4–10.9	Getahun [21]	2018	2.224(1.285–3.163)	80%
	2.5(1.1–5.7)	Gizachew [32]	2020		
	2.62(1.25–5.48)	Kelemu [33]	2023		
	1.9(1.04–3.47)	Gelila [41]	2020		

### Factors associated with ineffective breast-feeding technique

#### No formal education

Of the total included studies, four [20, 33, 35, 39–41] revealed the factors associated with ineffective breastfeeding techniques among breastfeeding mothers in Ethiopia (Table 4). Four studies found a significant association between lack of formal education and ineffective breastfeeding techniques among breastfeeding mothers. Of these the highest risk factors, AOR = 5.88(2.97, 7.31 [40] and the lowest risk factor AOR = 2.1(1.2, 3.50) [35] compared to those who were educated mothers (Table 4). Regarding heterogeneity test for no formal education, Galbraith plot showed heterogeneity, combining the results of four studies, the forest plot showed the overall estimate of AOR of no formal education was 3.433 (95%CI: (1.678–5.187); I^2^ = 72.2%; P = 0.0132).The I-squared (I^2^) and P-values also showed heterogeneity (S5 Fig).

Regarding publication bias for formal education, funnel plot analysis showed an asymmetrical distribution. We employed a leave-one-out sensitivity analysis to identify the potential source of heterogeneity in the pooled estimate of mothers with no formal education as a risk factor for ineffective breastfeeding techniques. The results of the sensitivity analysis showed that our findings were not dependent on a single study. Our pooled estimate of mothers with no formal education varied between 3.43 (95% CI, 1.67–5.18) and 4.1 (95%CI: 1.22–7.00) after deletion of a single study.

#### Being primipara mothers

Four studies found a significant association between primipara status and ineffective breastfeeding techniques among breastfeeding mothers. Of these the highest risk factors, AOR = 4.34(2.25, 8.36) [40] and lowest risk factor AOR = 1.8(1.0, 3.20) [39] compared to those who are multipara mothers (Table 4).

Regarding the heterogeneity test for being primipara, the Galbraith plot showed heterogeneity combining the results of four studies, and the forest plot showed that the overall estimate of AOR of Being primipara was 2.72 (95%CI: 1.81–3.64; I^2^ = 46.7%; P = 0.131).The I-squared (I^2^) and P-values also showed heterogeneity (S6 Fig). Regarding publication bias for primiparas, the funnel plot analysis showed an asymmetrical distribution. We employed a leave-one-out sensitivity analysis to identify the potential source of heterogeneity in the analysis of the pooled estimate of primipara mothers as a risk factor for ineffective breastfeeding technique. The results of this sensitivity analysis showed that our findings were not dependent on a single study. Our pooled estimate of being a primipara mother varied between 2.31(95%CI, 1.29–3.33) and 2.72 (95%CI: 1.81–3.64) after deletion of a single study.

#### Postnatal care

Four studies found a significant association between lack of postnatal care services and ineffective breastfeeding techniques among breastfeeding mothers. Of these the highest risk factors, AOR = 5.90(2.16, 15.90) [20] and lowest risk factor AOR = 1.94(1.7, 3.05) [41]. Comparing the results of the four studies, the forest plot showed that the overall estimate of AOR of ineffective breastfeeding techniques among breastfeeding mother was 1.84(95%CI: (1.35–2.32); I^2^ = 0%, P = 0.53). I^2^and P-value also showed homogeneity (S7 Fig and Table 4).

Regarding heterogeneity test, Galbraith plot showed homogeneity regarding test of publication bias a funnel plot showed an asymmetrical distribution. Egger’s regression test p-value was 0.002, which indicated the presence of publication bias (S8 Fig). Trim and fill analyses were performed, two studies were added, the total number of studies was 6, and the pooled estimate of AOR with low postnatal care service during breast feeding was 1.766 (95% CI: (1.29, 2.24); I^**2**^ = 0.0%; P = 0.516) (S9 Fig).

### Breastfeeding counseling

No breastfeeding counseling was significantly associated with ineffective breastfeeding techniques among the breastfeeding mothers. Of these the highest risk factors, AOR = 4.94(1.83, 13.36) [40] and lowest risk factor AOR = 2.3(1.4, 3.9) [39]. Compared with those who had been counseled about breastfeeding (Table 4). Combining the results of the three studies, the forest plot showed that the overall estimate of the AOR for having no breastfeeding counseling was 1.934 (95%C I: 1.23–2.63; I^2^ = 0%; P = 0.35). The (I^2^) and P values were also homogeneous (S10 Fig). Regarding the heterogeneity test, the Galbraith plot showed homogeneity, and regarding publication bias, the funnel plot showed a symmetrical distribution. In Egger’s regression test, the p-value was 0.214, indicating the absence of publication bias. We employed leave-one-out sensitivity analysis to identify the potential source of heterogeneity in the pooled estimate of breastfeeding counseling as a risk factor for ineffective breastfeeding techniques. The results of this sensitivity analysis showed that our findings were not dependent on a single study.

### Home delivery

Three studies found a significant association between home delivery and ineffective breastfeeding techniques among breastfeeding mothers. Of these the highest risk factors, AOR = 4.5(1.6–13.1) [39] and lowest risk factor AOR = 2.85 (1.22, 6.66) [33]. Compared with those who gave birth at the institution (Table 4). Regarding the heterogeneity test, the Galbraith plot showed heterogeneity, and combining the results of the three studies, the forest plot showed that the overall estimate of the AOR of home Delivery was 3.11(95% CI: 1.09, 5.16; I^2^ = 0%; P = 0.877). The I-squared (I^2^) and P-values also showed homogeneity (S11 Fig). Regarding publication bias, the funnel plot showed asymmetrical distribution. In Egger’s regression test, the p-value was 0.14, indicating that there was no publication bias. We employed a leave-one-out sensitivity analysis to identify the potential source of heterogeneity in the analysis of the pooled estimate of home delivery as a risk factor for ineffective breastfeeding among breastfeeding mothers. The results of the sensitivity analysis indicate that the findings were not dependent on a single variable.

### Breast problem

For the purposes of this study, the following were regarded as breast problems: breast discharge, breast pain, breast tumors, engorgement, breast lesions, and breast infections [35, 39]. Four studies found a significant association between breastfeeding problems and ineffective breastfeeding techniques among the mothers. Of these the highest risk factors, AOR = 4.02(1.40, 10.9) [20] and lowest risk factor AOR = 1.9 (1.04, 3.47) [41]. Compared with those who did not have breast problems during breastfeeding (Table 4). Regarding the heterogeneity test and combining the results of four studies, the forest plot showed that the overall estimate of AOR of Having breast problems during breastfeeding was 2.22(95%C I: 1.28, 3.16; I^2^ = 0.0%; P = 0.801). The I-squared (I^2^) and P-values also showed homogeneity (S12 Fig). Regarding publication bias, the funnel plot showed symmetrical distribution. Egger’s regression test showed a p-value of 0.013, the funnel plot showed an asymmetrical distribution, and Egger’s regression test indicated the presence of publication bias (S13 Fig). Due to the presence of publication bias, trim and fill analysis was performed; two studies were added, and the total number of studies was six. The pooled estimate of AOR Having breast problem during breast feeding was 2.03 (95%C I: (1.18, 2.87); I^2^ = 0.0%; P = 0.83).

## Discussion

This systematic review and meta-analysis assessed the prevalence of ineffective breastfeeding techniques and associated factors among breastfeeding mothers in Ethiopia. A total of 11 studies were included in the final analysis. All 11 studies had reported the ineffective breast-feeding techniques, and the pooled prevalence of ineffective breast-feeding techniques among breast feeding mothers was found to be 53.13% with 95% CI of (45.49–60.78%). These findings are consistent with those of studies conducted in West Denmark (48%), Cheluvamba Hospital in India (57%), and Libya (52%) [15, 42, 43]. but lower than that reported in a study conducted at West Bengal/Kolkata Hospital, India (69.7%) [18]. Possible reasons for these differences include variations in the nature of the study, as the Kolkata Hospital study was based in a single hospital, as well as differences in cultural practices and disparities in the study area, period, and sociodemographic variations between Ethiopia and India, which may have also contributed to the observed variation [18, 42].

This systematic review and meta-analysis revealed that using no formal education, being primipara, having low postnatal care counseling, home delivery, and having breast problems during breastfeeding were significant risk factors for increased prevalence of ineffective breastfeeding techniques among breastfeeding mothers in Ethiopia. Higher odds of ineffective breast feeding technique were observed in breast feeding women who have didn’t counseled about breast feeding technique during postnatal period [20].

The association between breastfeeding women who have not been counseled about breastfeeding and ineffective breastfeeding techniques during the postnatal period might be due to the fact that psychological support for breastfeeding mothers through early counselling and hands-on support for achieving proper techniques, particularly position and attachment.

According to this systematic review and meta-analysis, the odds of IBT were three times higher among women with no formal education than among those who attended secondary education and above. This finding is in line with the studies conducted in Indian East Delhi, West Bengal Kolkata hospital, Saudi Heraa general hospital, Bhaktapur district of Nepal and Sri Lanka. This might be due to the fact that uneducated women need much more time to adhere to and implement effective breastfeeding techniques. In addition, unschooled mothers may face difficulties in acquiring and observing health information regarding appropriate breastfeeding techniques [9, 18, 20, 43–45].

In this systematic review, the likelihood of IBT was almost three times higher among primiparous mothers than among multiparous mothers. This is in line with studies conducted in India, Denmark, Cheluvamba Hospital of India, and Libya. This might be due to a shortage of information, skills, and experience in breastfeeding techniques. Moreover, multiparous women are more likely to have gained childrearing experience (including feeding techniques) from previous pregnancies [9, 15, 20, 34, 43].

In this systematic review, the odds of IBT were twice as high among participants with breast problems compared with their complements. This is in line with studies conducted in Libya [15]. This might be due to the fact that mothers with breast problems may have severe pain that hinders them to apply breastfeeding techniques. In addition, it is difficult to correctly attach the infant’s mouth with engorged and cracked nipples because of distension and edema of the nipple. Mothers who did not receive counseling on breastfeeding techniques after delivery were almost twice as likely to exhibit IBT as those who received adequate information. This is consistent with studies conducted in rural areas of Nagpur district and India [19, 20]. Likewise, the odds of IBT were two times higher among participants who did not receive counseling about breastfeeding techniques during pregnancy than among their counterparts. This finding is consistent with those of previous studies conducted in Libya. This might be due to the fact that adequate counseling about breastfeeding during pregnancy and the postpartum period are imperative to achieving effective breastfeeding techniques. Moreover, the psychological support provided to lactating mothers has a significant effect on breastfeeding techniques enhancement [15].

The odds of IBT were three times higher among participants who delivered at home than among those who delivered in the hospital. This is in line with the studies conducted in the Bhaktapur district of Nepal. A possible reason is that women who deliver in the hospital are likely to receive continuous psychological and hands-on support to realize effective breastfeeding techniques [45].

### Strength and limitations

This study has several strengths. First, a predetermined methodology was used for the search strategy and data abstraction. Internationally recognized instruments were utilized as a critical appraisal system to evaluate the quality of individual investigations. Second, to determine the small study effect and risk of heterogeneity, we used subgroup and sensitivity analyses depending on the study location, study sample size, and publication year. Although this systematic review and meta-analysis aimed to estimate the national prevalence of ineffective breastfeeding techniques and their associated factors, it is not representative of all regions, as data were not found in some regions of the country.

## Conclusion and recommendation

In Ethiopia, the prevalence of ineffective breastfeeding techniques among mothers remains high. There was no formal education, being primipara, low postnatal care counselling, home delivery, and having a breast problem during breastfeeding as independent potential predictors of ineffective breastfeeding techniques among Ethiopian breastfeeding mothers. To prevent these risk factors, adequate intervention on potential determinants such as educating and empowering mothers, providing breastfeeding counseling, and supporting institutional delivery is recommended.

## Supporting information

S1 Fig Subgroup analysis of the prevalence of ineffective breastfeeding techniques among breastfeeding mothers in Ethiopia by region. (DOCX)

S2 Fig Subgroup analysis of the prevalence of ineffective breastfeeding techniques among breastfeeding mothers in Ethiopia by sample size. (DOCX)

S3 Fig Subgroup analysis of the prevalence of ineffective breastfeeding technique among breastfeeding mothers in Ethiopia by year of publication. (DOCX)

S4 Fig Sensitivity analysis of a pooled estimate of ineffective breastfeeding technique among breastfeeding mothers in Ethiopia from 2010 up to 2023. (DOCX)

S5 Fig Forest plot showing the pooled estimate of AOR for no formal education as a predictor of ineffective breastfeeding technique among breastfeeding mothers in Ethiopia from 2010 up to 2023. (DOCX)

S6 Fig Forest plot showing the pooled estimate of AOR for being primipara as a predictor of ineffective breastfeeding technique among breastfeeding mothers in Ethiopia from 2010 up to 2023. (DOCX)

S7 Fig Forest plot showing the pooled estimate of AOR for postnatal care as a predictor of ineffective breastfeeding technique among breastfeeding mothers in Ethiopia from 2010 up to 2023. (DOCX)

S8 Fig Sensitivity analysis of a pooled estimate of AOR for using postnatal care services as a predictor variable for ineffective breastfeeding technique among breastfeeding mothers in Ethiopia from 2010 up to 2023. (DOCX)

S9 Fig Trim and fill analysis for pooled estimate of AOR for using postnatal care service as a predictor variable for ineffective breastfeeding technique among breastfeeding mothers in Ethiopia from 2010 up to 2023. (DOCX)

S10 Fig Forest plot showing the pooled estimate of AOR for breast feed counseling as a predictor of ineffective breastfeeding technique among breastfeeding mothers in Ethiopia from 2010 up to 2023. (DOCX)

S11 Fig Forest plot showing the pooled estimate of AOR for home delivery as a predictor of ineffective breast-feeding technique among breastfeeding mothers in Ethiopia from 2010 up to 2023. (DOCX)

S12 Fig Forest plot showing the pooled estimate of AOR for breast problem as a predictor of ineffective breast-feeding technique among breastfeeding mothers in Ethiopia from 2010 up to 2023. (DOCX)

S13 Fig Sensitivity analysis of pooled estimate of AOR for using having breast problem as a predictor variable for ineffective breast-feeding technique among breastfeeding mothers in Ethiopia from 2010 up to 2023. (DOCX)

S1 Table Different databases were searched to find articles on ineffective breastfeeding techniques and associated factors in Ethiopia. (DOCX)

S1 File Searching terms used for Google Scholar. (DOCX)

S2 File The searching terms for PubMed and web of science. (DOCX)

S1 Data Minimal anonymized dataset excel format. (XLSX)

## Abbreviations

CI: Confidence Interval
OR: Odds Ratio
WHO: World Health Organization
DHS: Demographic and Health Surveys
EDHS: Ethiopian Demographic and Health Survey
AOR: Adjusted odds ratio
ARTI: IBT: ineffective breast-feeding technique
PNC: post-natal care

## References

[1] Wambach K. and Spencer B., Breastfeeding and human lactation. 2019: Jones & Bartlett Learning.

[2] Yasmeen T., et al., Benefits of breastfeeding for early growth and long term obesity: A summarized review. International Journal of Medical Science and Diagnosis Research (IJMSDR), 2019. 3(1).

[3] As’ ad S. and Idris I., Relationship between Early Breastfeeding Initiation and Involution Uteri of Childbirth Mothers in Nenemallomo Regional Public Hospital and Arifin Nu’mang Public Regional Hospital of SidenrengRappang Regency in 2014. Indian Journal of Public Health Research & Development, 2019. 10(4).

[4] González-Jiménez E., Breastfeeding and reduced risk of breast cancer in women: a review of scientific evidence. Selected Topics in Breastfeeding, R. Mauricio Barría P, IntechOpen, 2018: p. 55–64.

[5] Modugno F., et al., Breastfeeding factors and risk of epithelial ovarian cancer. Gynecologic oncology, 2019. 153(1): p. 116–122. doi: 10.1016/j.ygyno.2019.01.017 30686553 PMC6558958

[6] Duan X., Wang J., and Jiang X., A meta-analysis of breastfeeding and osteoporotic fracture risk in the females. Osteoporosis International, 2017. 28: p. 495–503. doi: 10.1007/s00198-016-3753-x 27577724

[7] Rajaei S., et al., Breastfeeding duration and the risk of coronary artery disease. Journal of Women’s Health, 2019. 28(1): p. 30–36. doi: 10.1089/jwh.2018.6970 30523760 PMC6422010

[8] Lancet T., Breastfeeding: achieving the new normal. The Lancet, 2016. 387(10017): p. 404. doi: 10.1016/S0140-6736(16)00210-5 26869549

[9] Parashar M., et al., Breastfeeding attachment and positioning technique, practices, and knowledge of related issues among mothers in a resettlement colony of Delhi. ICAN: Infant, Child, & Adolescent Nutrition, 2015. 7(6): p. 317–322.

[10] Radzewicz E., et al., Breastfeeding as an important factor of reduced infants’ infection diseases. Progress in Health Sciences, 2018. 8: p. 70–74.

[11] Infant W., young child feeding: Model chapter for textbooks for medical students and allied health professionals. Mt. Res. Dev. Geneva, Switzerland: WHO Library Cataloguing-inPublication Data, 2009. 23905206

[12] Sankar M.J., et al., Optimal breastfeeding practices and infant and child mortality: a systematic review and meta‐analysis. Acta paediatrica, 2015. 104: p. 3–13. doi: 10.1111/apa.13147 26249674

[13] Santos K.J.d.S., et al., Prevalence and factors associated with cracked nipples in the first month postpartum. BMC pregnancy and childbirth, 2016. 16: p. 1–8. 27496088 10.1186/s12884-016-0999-4 PMC4975913

[14] EPHI, I., Ethiopian public health Institute (EPHI)[Ethiopia] and ICF. Ethiopia Mini Demographic and Health Survey 2019: Key Indicators, 2019.

[15] Goyal R.C., et al., Breastfeeding practices: positioning, attachment (latch-on) and effective suckling–a hospital-based study in Libya. Journal of Family and Community Medicine, 2011. 18(2): p. 74. doi: 10.4103/2230-8229.83372 21897915 PMC3159232

[16] NICEF, W., World Bank, N DESA/Population Division. Levels and Trends in Child Mortality. UNI CEF. 2019. 2019.

[17] Joshi H., Magon P., and Raina S., Effect of mother–infant pair’s latch-on position on child’s health: A lesson for nursing care. Journal of family medicine and primary care, 2016. 5(2): p. 309. doi: 10.4103/2249-4863.192373 27843833 PMC5084553

[18] Dasgupta U., et al., Breastfeeding practices: positioning, attachment and effective suckling—a hospital based study in West Bengal/Kolkata. Indian J Mater Child Health, 2013. 15: p. 1–11.

[19] Thakre S.B., et al., The Breastfeeding Practices: The Positioning and Attachment Initiative Among the Mothers of Rural Nagpur. Journal of Clinical & Diagnostic Research, 2012. 6(7).

[20] Tiruye G., et al., Breastfeeding technique and associated factors among breastfeeding mothers in Harar city, Eastern Ethiopia. International breastfeeding journal, 2018. 13(1): p. 1–9. 10.1186/s13006-018-0147-z PMC5791732 29434650

[21] Kishore M.S.S., Kumar P., and Aggarwal A.K., Breastfeeding knowledge and practices amongst mothers in a rural population of North India: a community-based study. Journal of tropical pediatrics, 2009. 55(3): p. 183–188. doi: 10.1093/tropej/fmn110 19074494

[22] Singh S., et al., Breastfeeding practices in occupational castes of Sunsari district of Nepal. Health Renaissance, 2013. 11(3): p. 219–223.

[23] Organization, W.H., Protecting, promoting and supporting breastfeeding in facilities providing maternity and newborn services: the revised Baby-friendly Hospital initiative: 2018 implementation guidance: frequently asked questions. 2020.

[24] Peters M.D., et al., The Joanna Briggs Institute reviewers’ manual 2015: methodology for JBI scoping reviews. 2015.

[25] Muche A.A., et al., Prevalence and determinants of risky sexual practice in Ethiopia: systematic review and meta-analysis. Reproductive health, 2017. 14: p. 1–11. 28877736 10.1186/s12978-017-0376-4 PMC5588747

[26] Safayi B.L., Assimamaw N.T., and Kassie D.G., Breastfeeding technique and associated factors among lactating mothers visiting Gondar town health facilities, Northwest Ethiopia: observational method. Italian Journal of Pediatrics, 2021. 47(1): p. 1–10. 34641916 10.1186/s13052-021-01158-6 PMC8507121

[27] Organization W.H., Breastfeeding counselling: a training course. 1993, World Health Organization.

[28] Borenstein M., et al., A basic introduction to fixed‐effect and random‐effects models for meta‐analysis. Research synthesis methods, 2010. 1(2): p. 97–111. doi: 10.1002/jrsm.12 26061376

[29] Higgins J.P., et al., Measuring inconsistency in meta-analyses. Bmj, 2003. 327(7414): p. 557–560. doi: 10.1136/bmj.327.7414.557 12958120 PMC192859

[30] Ioannidis J.P., Interpretation of tests of heterogeneity and bias in meta‐analysis. Journal of evaluation in clinical practice, 2008. 14(5): p. 951–957. doi: 10.1111/j.1365-2753.2008.00986.x 19018930

[31] Higgins J.P. and Thompson S.G., Quantifying heterogeneity in a meta‐analysis. Statistics in medicine, 2002. 21(11): p. 1539–1558. doi: 10.1002/sim.1186 12111919

[32] Phua Q.S., et al., Systematic analysis of publication bias in neurosurgery meta-analyses. Neurosurgery, 2022. 90(3): p. 262–269. doi: 10.1227/NEU.0000000000001788 35849494

[33] Asmamaw D.B., et al., Effective breastfeeding technique and associated factors among lactating mothers in Gidan District, North-East, Ethiopia: a community-based cross-sectional study. BMJ open, 2022. 12(7): p. e059518. doi: 10.1136/bmjopen-2021-059518 35858723 PMC9305837

[34] Degefa N., et al., Breast feeding practice: positioning and attachment during breast feeding among lactating mothers visiting health facility in Areka Town, Southern Ethiopia. International journal of pediatrics, 2019. 2019. doi: 10.1155/2019/8969432 31080479 PMC6475560

[35] Desse H., Assebe T., and Girma S., MAGNITUDE OF INEFFECTIVE BREASTFEEDING TECHNIQUES AND ASSOCIATED FACTORS AMONG LACTATING MOTHERS VISITING PUBLIC HOSPITALS IN SOUTH GONDAR ZONE, NORTHWEST, ETHIOPIA. 2022, Haramaya University.

[36] HIRUT Y., BREASTFEEDING TECHNIQUE AND ASSOCIATED FACTORS AMONG PRIMI-PARA MOTHERS IN BASONA WERANA DISTRICT, NORTH SHEWA, ETHIOPIA, 2022. 2022.

[37] Tamiru D. and Jisha H., Breastfeeding skills in Arba Minch Zuria: the positioning and attachment initiatives. International Journal of Nursing and Midwifery, 2017. 9(4): p. 46–52.

[38] WETET T., BREAST FEEDING TECHNIQUES AND ASSOCIATED FACTORS AMONG PRIMIPAROUS MOTHERS IN SOUTH ACHEFER WOREDA, WEST GOJJAM ZONE, AMHARA REGION, ETHIOPIA, 2021. 2021.

[39] Yilak G., et al., Prevalence of ineffective breastfeeding technique and associated factors among lactating mothers attending public health facilities of South Ari district, Southern Ethiopia. PloS one, 2020. 15(2): p. e0228863. doi: 10.1371/journal.pone.0228863 32045451 PMC7012449

[40] Alemie K., et al., Ineffective breastfeeding techniques and associated factors among breastfeeding mothers who gave birth in the last 6 months in Sinan Woreda, Northwest Ethiopia. Journal of Public Health Research, 2023. 12(2): p. 22799036231181184. 37440796 10.1177/22799036231181184 PMC10334002

[41] GELILA MEKURIY Z.A.e.a., BREAST FEEDING TECHNIQUES AND ASSOCIATED FACTORS AMONG PRIMI-PARA MOTHERS IN PUBLIC HEALTH CENTERS, ADDIS ABABA, ETHIOPIA. Addis Ababa university repository, 2020. 251.

[42] Kronborg H. and Væth M., How are effective breastfeeding technique and pacifier use related to breastfeeding problems and breastfeeding duration? Birth, 2009. 36(1): p. 34–42. doi: 10.1111/j.1523-536X.2008.00293.x 19278381

[43] Nagendra K., et al., Evaluation of breast feeding techniques among postnatal mothers and effectiveness of intervention: Experience in a tertiary care centre. Sri Lanka Journal of Child Health, 2017. 46(1).

[44] El-Khedr S.M. and Lamadah S.M., Knowledge, attitude and practices of Saudi women regarding breastfeeding at Makkah al Mukkaramah. J Biol Agriculture Health Care, 2014. 4: p. 56–65.

[45] Paudel D.P. and Giri S., Breast feeding practices and associated factors in Bhaktapur District of Nepal: A community based cross-sectional study among lactating mothers. Journal of the Scientific Society, 2014. 41(2): p. 108–113.

