# Supplementary material for: Prevalence of ineffective breastfeeding techniques and its associated factors among breastfeeding mothers in Ethiopia: A systematic review and meta-analysis
Source: PLoS One. 2024 Jun 13;19(6):e0303749. doi: 10.1371/journal.pone.0303749 (PMC11175424; doi:10.1371/journal.pone.0303749)
Supplement: S3 Fig — (DOCX) [file pone.0303749.s003.docx]

Supplementary Figure 3: Subgroup analysis of the prevalence of ineffective breastfeeding technique among breastfeeding mothers in Ethiopia by year of publication.
