# Supplementary material for: Prevalence of ineffective breastfeeding techniques and its associated factors among breastfeeding mothers in Ethiopia: A systematic review and meta-analysis
Source: PLoS One. 2024 Jun 13;19(6):e0303749. doi: 10.1371/journal.pone.0303749 (PMC11175424; doi:10.1371/journal.pone.0303749)
Supplement: S1 Table — (DOCX) [file pone.0303749.s014.docx]

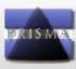
PRISMA (preferred items for systematic review and meta-analysis) checklist

You must report the page number in your manuscript where you consider each of the items listed in this checklist, if you have not included this information either revise your manuscript accordingly before submitting or note N/A

| Section and  Topic | Item  # | Checklist item | Report on page number |
| --- | --- | --- | --- |
| TITLE | | |  |
| Title | 1 | Identify the report as a systematic review, meta-analysis, or both. | 1 |
| ABSTRACT | | |  |
| Structured summery | 2 | Provide structured summery including as applicable : background, objective, data sources, study eligibility criteria, participants and interventions, and study  Appraisal and synthesis method : result, limitation, conclusion and implication of key findings | 2 |
| INTRODUCTION | | |  |
| Rationale | 3 | Describe the rationale for the review in the context of existing knowledge. | 3 |
| Objectives | 4 | Provide an explicit statement of the objective(s) or question(s) the review addresses. | 3 |
| METHODS | | |  |
| Protocol and registration | 5 | Indicate if review protocol exists, if and where it can be assessed (eg , web address), and if available provide registration information including registration number | 4 |
| Eligibility criteria | 7 | Specify study characteristics, (eg PICO, length of follow up) and report characteristics (eg , year considered, language , publication status ) used as a  criteria for eligibility , giving rational | 5 |
| Information sources | 8 | .describe all information sources (eg database with date of coverage , contact with study authors to identify additional studies) in the search and date last searched. | 5 |
| Data collection process | 9 | Specify the methods used to collect data from reports, including how many reviewers collected data from each report, whether they worked independently, any processes for obtaining or confirming data from study investigators, and if applicable, details of automation tools used in the process. | 5 |
| Search | 10 | Present full electronic search strategy for at least one database including any limit used, such that it could be repeated | 6 |
| Study selection | 11 | State the process for selecting studies (eg: screening, eligibility included in systematic review, and , if applicable included in meta-analysis | 6 |
| Data collection process | 12 | Describe method of data extraction from reports (eg, piloted form, independently, in duplicates and any process obtaining and confirm data from investigators | 6 |
| Data items | 13 | List and define all variables for which data were sought ( eg, PICO funding sources ) any assumption and simplification made | 6 |
| Risk of bias in individual studies | 13f | Describe the method used for assessing risk of bias of individual studies (including specification of whether this was done as a study or outcome level, and how this information is to be used in any data synthesis | 6 |

| Section and  Topic | Item  # | Checklist item | Report on page number |
| --- | --- | --- | --- |
| RESULTS | | |  |
| Study selection | 15 | Give number of study screened , assessed for eligibility and included in the review with reason for exclusion at each stage , ideally with flow diagram | 7 |
| Studies characteristics | 16 | For each studies, present characteristics for which data were extracted (eg, study size, PICO, follow-up period and provide the citation | 7 |
| Risk of bias within studies | 17 | Present data on risk of bias of which study and if available, any outcome level assessment | 8 |
| Results of individual studies |  | For all outcome considered (benefit or harm), present, for each study simple summary of data for each intervention group (b) effect estimate and confidence interval and ideally with forest pilot | 10 |
| Synthesis of result | 19 | Present results of each meta-analysis done , including confidence interval and measure of consistency | 12 |
| DISCUSSION | | |  |
| Summary of evidence | 21 | Summarize the main finding including the strength of evidence for the main outcome, consider their relevance to key groups (eg, health care provider, users and policy makers | 13 |
| Limitations | 22 | Discussion limitation at study and outcome level (eg, risk of bias) and at reviewer level (eg, incomplete retrieval of identified research and reporting bias | 15 |
| Conclusion | 23 | Provide general interpretation of the result in the context of other evidence, and implication for future research | 16 |
| Funding | 24 |  | 16 |

From: Page MJ, McKenzie JE, Bossuyt PM, Boutron I, Hoffmann TC, Mulrow CD, et al. The PRISMA 2020 statement: an updated guideline for reporting systematic reviews. BMJ 2021;372:n71. doi: 10.1136/bmj.n71

For more information, visit:<http://www.prisma-statement.org/>
