# Supplementary material for: Prevalence of ineffective breastfeeding techniques and its associated factors among breastfeeding mothers in Ethiopia: A systematic review and meta-analysis
Source: PLoS One. 2024 Jun 13;19(6):e0303749. doi: 10.1371/journal.pone.0303749 (PMC11175424; doi:10.1371/journal.pone.0303749)
Supplement: S2 File — (DOCX) [file pone.0303749.s016.docx]

Medical Subject Headings (MeSH) terms and keywords were used to conduct the search.

"(Prevalence OR (Prevalence[MeSH Terms]) magnitude OR magnitude [MeSH Terms]) or epidemiology) OR (epidemiology [MeSH Terms]) AND (causes OR (causes [MeSH Terms]) determinants OR (determinants [MeSH Terms]) (related factors) OR (related factors [MeSH Terms]) OR predictors OR (predictors[MeSH Terms]) OR (risk factors) OR (risk factors [MeSH Terms]) OR (poor breastfeeding techniques OR (poor breastfeeding techniques [MeSH Terms]) (ineffective breastfeeding techniques OR (ineffective breastfeeding techniques [MeSH Terms]) OR (position, attachment, and suckling OR (position, attachment, and suckling [MeSH Terms]) OR (effective breastfeeding techniques) OR (effective breastfeeding techniques [MeSH Terms]) AND (Ethiopia)"
